# Prevalence of hepatitis C virus (HCV) variants resistant to NS5A inhibitors in naïve patients infected with HCV genotype 1 in Tunisia

**DOI:** 10.1186/s12985-015-0318-0

**Published:** 2015-06-06

**Authors:** Jameleddine Aissa Larousse, Pascale Trimoulet, Patricia Recordon Pinson, Brigitte Tauzin, Mohamed Mssadak Azzouz, Nabyl Ben Mami, Imed Cheikh, Henda Triki, Hervé Fleury

**Affiliations:** LR11-IPT-09, Epidémiologie et diversité génétique des virus hépatiques et entériques humains, Institut Pasteur de Tunis, 13 Place Pasteur, 1002 Tunis Belvédère, Tunisia; CNRS-UMR 5234, Microbiologie fondamentale et Pathogénicité, University of Bordeaux 2, Bordeaux, France; Virology Laboratory, Bordeaux University Hospital, Bordeaux, France; Department of Gastroenterology, Hospital of Tahar Maamouri, Nabeul, Tunisia; Department of Gastroenterology B, La Rabta Hospital, Tunis, Tunisia; Department of Gastroenterology, Habib Bougatfa Hospital, Bizerte, Tunisia

**Keywords:** Hepatitis C virus, Non-structural protein 5A, Direct-acting antivirals, Resistance mutations

## Abstract

**Background:**

Hepatitis C virus (HCV) non-structural protein 5A (NS5A) inhibitors have been recently developed to inhibit NS5A activities and have been approved for the treatment of HCV infection. However the drawback of these direct acting antivirals (DAAs) is the emergence of resistance mutations. The prevalence of such mutations conferring resistance to HCV-NS5A inhibitors before treatment has not been investigated so far in the Tunisian population. The aim of this study was to detect HCV variants resistant to HCV-NS5A inhibitors in hepatitis C patients infected with HCV genotype 1 before any treatment with NS5A inhibitors.

**Methods:**

Amplification and direct sequencing of the HCV NS5A region was carried out on 112 samples from 149 untreated patients.

**Results:**

In genotype 1a strains, amino acid substitutions conferring resistance to NS5A inhibitors (M28V) were detected in 1/7 (14.2 %) HCV NS5A sequences analyzed. In genotype 1b, resistance mutations in the NS5A region (R30Q; L31M; P58S and Y93H) were observed in 17/105 (16.2 %) HCV NS5A sequences analyzed. R30Q and Y93H (n = 6; 5.7 %) predominated over P58S (n = 4; 3.8 %) and L31M (n = 3; 2.8 %).

**Conclusions:**

Mutations conferring resistance to HCV NS5A inhibitors are frequent in treatment-naïve Tunisian patients infected with HCV genotype 1b. Their influence in the context of DAA therapies has not been fully investigated and should be taken into consideration.

## Background

Hepatitis C virus (HCV) infection is the major cause of chronic liver disease which can progress to cirrhosis and hepatocellular carcinoma over the course of 20–30 years [1]. Globally HCV has infected 180 million people, approximately 3 % of the world’s population [2]. It is an enveloped positive-strand RNA virus and is the only member of the *Hepacivirus* genus of the *Flaviviridae* family [3]. Based on phylogenetic analysis, HCV has been classified into six different genotypes and diverse subtypes [4].

Tunisia is one of the countries with low endemicity for HCV. The prevalence of HCV infection is less than 1 % [5, 6] with an average prevalence of 80 % of subtype 1b [7, 8]. The current standard therapy in Tunisia consists of pegylated alpha interferon (PEG-IFNα) combined with ribavirin (RBV), a nucleoside analogue, for 24–48 weeks [9]. This treatment is associated with a high long-term response rate in patients infected with HCV genotype 2 and 3, whereas a sustained virologic response (SVR) can be achieved only in 50 % of patients infected with HCV genotype 1 [10–12].

Recently, direct-acting antivirals (DAAs) that target specific viral proteins of the HCV life cycle, have been developed and some of them have already been approved in some countries for the treatment of HCV infection in combination with PEG-IFNα/RBV as well as in IFN-free regimens [13]. These molecules include a range of non-structural (NS) 3/NS4A protease, NS5B polymerase and NS5A inhibitors [14].

NS5A inhibitors target the domain I of NS5A protein and have been shown to block phosphorylation of NS5A, which is essential for viral RNA replication and inhibits the assembly and release of viral particles [15, 16]. These inhibitors have been tested in clinical trials. Data for first-generation NS5A inhibitors such as daclatasvir (DCV), ledipasvir (LDV) and ombitasvir (OBV) are available. The effectiveness of DCV has been studied in combination with PEG-IFNα/RBV among naïve patients. For a treatment lasting of 24 or 48 weeks, SVR rates were 59 and 58 % in patients with subtype 1a, 78 and 87 % in patients with subtype 1b, and 67 and 100 % in patients with HCV genotype 4, respectively [15]. In naïve patients, the combination of sofosbuvir (SOF)/LDV, administered for 12 weeks with or without RBV, has been associated with SVR rates of 95 % [17, 18]. Similar response rates were obtained with treatment durations of only 8 weeks in patients with a fibrosis score of F0-F1 [18]. In patients failing a first-line therapy with the association PEG-IFNα/RBV/first-generation protease inhibitors, SVR rates with SOF/LDV were 95 and 99 % after 12 and 24 weeks of treatment respectively [17]. OBV was evaluated in vivo in a 3-day monotherapy study in 12 HCV genotype 1-infected patients at 5, 25, 50 and 200 mg dosed once daily and showed significant efficiency [19].

The rapid replication rate of HCV and the low fidelity of its polymerase result in a sequence variation in the HCV population, leading to a quasi-species and the potential selection of drug-resistant mutations [14]. The efficacy of DAA is limited by the presence of these mutations resulting in amino acid substitutions within the targeted proteins which affect viral sensitivity to these compounds [14]. Monotherapy studies with NS5A inhibitors have revealed the rapid emergence of resistance mutations to these drugs [20]. In vitro or in vivo, five major positions of mutations in the NS5A HCV protein (28; 30; 31; 58 and 93) have been reported to be associated with different levels of resistance to NS5A inhibitors [14]. It has been demonstrated that HCV NS5A inhibitors should be used in combination regimens potent enough to prevent the emergence of resistance mutations [21].

Variants conferring resistance to NS5A inhibitors exist naturally within HCV quasispecies populations in the absence of any previous exposure to these drugs [16, 22–24] while they are frequently selected at failure with treatment including HCV NS5A inhibitors [21].

HCV NS5A inhibitors are not yet available in Tunisia, but it is very important to determine the natural prevalence of such substitutions conferring resistance to NS5A inhibitors before their introduction in the country for more extensive treatment of HCV infection.

The aim of this study was to investigate the natural prevalence of mutations conferring resistance to NS5A inhibitors in clinical strains from treatment-naïve Tunisian patients infected with HCV genotype 1.

## Results

### Baseline characteristics of the patients

All patients were infected with HCV genotype 1 (1b: 142; 1a:7), all of them were naïve of anti-HCV treatment and none was co-infected with hepatitis B virus (HBV) or human immunodeficiency virus (HIV). Their median age was 56 years. Forty-five patients (30 %) out of 149 were male and 104 patients (70 %) were female. The median HCV load was 1,955,625 IU/ml [range 9140-16,800,000 IU/ml]. 49 % of patients had liver cirrhosis.

### Prevalence of NS5A inhibitor resistance mutations

The NS5A gene was successfully sequenced in 112 out of 149 (75.1 %) samples that were amplified by nested PCR. Among the 112 NS5A sequences obtained, 7 sequences (6.2 %) were HCV genotype 1a and 105 (93.8 %) HCV genotype 1b.

Sequencing and alignments of the 112 NS5A sequences allowed the identification of amino acid substitutions implicated in resistance to HCV NS5A inhibitors (Table 1).

**Table 1:** Amino acid substitutions in NS5A region of treatment-naïve patients infected with HCV genotypes 1a (n = 7) and 1b (n = 105)

NS5A residues		Resistance mutations		Prevalence of observed resistance mutations (%)		DAAs
Genotypes		Genotypes		Genotypes		
1a	1b	1a	1b	1a	1b	
	L23		F			Daclatasvir
M28	L28	T/V	M/V	V (1/7; 14.2 %)		Daclatasvir Ombitasvir
Q30	R30	H/E/R/K	Q		Q (6/105; 5.7 %)	Daclatasvir Ombitasvir
L31	L31	M/V	F/M/V		M (3/105; 2.8 %)	Daclatasvir Ledipasvir Ombitasvir
P32	P32	L	L			Daclatasvir
	P58		S		S (4/105; 3.8 %)	Daclatasvir
Y93	Y93	C/H/N	C/H/N		H (6/105; 5.7 %)	Daclatasvir Ledipasvir Ombitasvir

Data interpreted according to Krishnan et al. [19] Paolucci et al. [24] and Fridell et al. [27]

Amino acid substitutions conferring resistance to HCV NS5A inhibitors were detected in both subtypes 1a and 1b strains.

In subtype 1a strains, amino acid substitutions conferring resistance to HCV NS5A inhibitors were observed in 1 sequence out of 7 (14.2 %). This sequence showed the M28V substitution associated with very low-level resistance to DCV and displayed 58-fold resistance to OBV.

In genotype 1b strains, amino acid substitutions associated with resistance to HCV NS5A inhibitors were observed in 17/105 (16.2 %) sequences analyzed. Three sequences (3/105; 2.8 %) exhibited the substitution L31M and six sequences (6/105; 5.7 %) showed the Y93H variant. These mutations are considered as primary resistance mutations. The L31M substitution is known to confer 3-fold resistance to DCV and 2.09-fold resistance to LDV. The Y93H mutation is known to confer 24-fold resistance to DCV, 77-fold resistance to OBV and very high-level resistance to LDV yielding 1319-fold resistance. Six sequences (6/105; 5.7 %) exhibited the R30Q substitution and P58S was present in four sequences (4/105; 3.8 %). These two substitutions are known as secondary resistance mutations that increase the resistance of primary mutations but do not confer resistance by themselves.

None of the 112 NS5A sequences from either subtype contained the P32L mutation, which is known to be a primary resistant variant associated with high-level resistance to HCV NS5A inhibitors.

Combinations of viral variants in the NS5A region were rare. No combinations of viral variants were observed in genotype 1a. Among the 105 NS5A 1b sequences analyzed, only two sequences (2/105; 1.9 %) showed combinations of two resistant variants R30Q + Y93H. When present on the same genome, this combination is known to confer 284-fold resistance to OBV.

## Discussion

In this study, the first in the Tunisian population, 149 treatment-naïve patients infected with HCV genotype 1 were evaluated to determine the natural prevalence of HCV variants resistant to NS5A inhibitors. The sequencing technology used was the Sanger method which is known to allow detection of dominant variant populations above 20 %. We cannot eliminate minor variant populations that might have been detected by ultra-deep pyro-sequencing, whose threshold for detecting minor variants is estimated to be fairly lower than the Sanger threshold.

Baseline identification of pre-existing mutations resistant to anti-HCV inhibitors in treatment-naïve patients is essential for defining new DAAs therapies [24]. Some studies have reported that all amino acid substitutions associated with resistance to HCV NS5A inhibitors are located in the N-terminal region of NS5A [25, 26]. Of note, some studies have shown that the majority of resistance mutations observed in vivo are at positions M28, Q30, L31 and Y93 for HCV genotype 1a and at positions L31 and Y93 for genotype 1b [27, 28].

In this work some resistance mutations conferring resistance to HCV NS5A inhibitors such as DCV, LDV and OBV were observed. One out of 7 (14.2 %) patients with subtype 1a and 17 out of 105 (16.2 %) with subtype 1b showed amino acid substitutions within the NS5A inhibitor resistance sites. In this study, the number of patients infected with HCV genotype 1b was very large compared to the number of patients infected with HCV genotype 1a, which is due to the high 80 % prevalence of genotype 1b in Tunisia [6–8]. In view of the low number of 1a isolates studied, it is not possible to make a definitive comparison of the resistance mutation to NS5A inhibitors between both subtypes.

Some studies have described the natural prevalence of substitutions conferring resistance to HCV inhibitors in patients naïve to treatment with NS5A inhibitors. In Italy, Paolucci et al. reported a frequency of 12.5 % and 53.3 % of substitutions involved in resistance to HCV NS5A inhibitors in DAA treatment-naïve patients infected with genotype 1a and 1b, respectively [24]. Suzuki et al. reported 11.2 % of mutations L31M/V and Y93H conferring resistance to HCV NS5A inhibitors in treatment-naïve patients infected with HCV genotype 1b in Japan [23]. In the present study we considered all mutations conferring resistance to HCV NS5A inhibitors for genotype 1b and our results are close to those provided by Suzuki et al.

In subtype 1b strains, the primary resistance mutations L31M conferring low-level resistance to DCV [27] and Y93H conferring high-level resistance to DCV [27] and OBV [19] and very high-level resistance to LDV [24] were observed in three (2.8 %) and six sequences (5.7 %), respectively. A previous study demonstrated that the prevalence of these two key mutations associated with resistance to NS5A inhibitors ranges from 6 to 12 % among genotype 1b sequences [29]. The prevalence of these mutations may reduce the barrier of resistance and influence virologic response [29]. Bartels et al. found a prevalence of 6.2 % and 3.7 % of the resistant variants L31M and Y93H, respectively in patients infected with subtype 1b [30]. These results are close to those observed in the present study. Substitutions associated with resistance to NS5A inhibitors have been associated with low rates of virologic response in some DCV-based regimens [29]. However, Suzuki et al. demonstrated in a phase II study that many patients with pre-existing resistance mutations to NS5A inhibitors were cured of their chronic HCV infection [31].

Moreover, other substitutions like R30Q (5.7 %) and P58S (3.8 %) were found. These variants are known as secondary resistance mutations that increase the resistance of primary mutations but do not confer resistance by themselves [25]. None of the 112 NS5A sequences analyzed for either subtype showed any substitution at position 32, which is known to confer high-level resistance to DCV for subtype 1a [25] and a moderate level of resistance for subtype 1b [25]. These results are similar to those in Bartels’ study [30].

In subtype 1a, a primary resistance mutation in the NS5A region M28V conferring low-level resistance to DCV [25] and high-level resistance to OBV [19] was observed in one sequence (14.2 %). One study has demonstrated that DCV is characterized by a low genetic barrier of resistance, notably for genotype 1a in which the resistance to the drug can be acquired through a single mutation [29].

In conclusion, the present study in treatment-naïve Tunisian patients infected with HCV genotype 1b demonstrates a significant proportion of substitutions conferring resistance to HCV NS5A. Their influence in the context of DAAs therapies has not yet been fully investigated and should be taken into consideration. Further studies are needed to determine the impact of the NS5A baseline resistance mutations on the treatment outcome in clinical trials investigating combination therapies of NS5A inhibitors with other classes of DAAs, although it can be predicted that combination therapies would raise the resistance barrier and reduce the importance of natural substitutions at resistance positions to NS5A inhibitors.

## Materials and methods

### Patients

The current study included 149 Tunisian patients infected with HCV genotype 1 who were referred to the gastroenterology department in three Tunisian University Hospitals between 2009 and 2011: Habib Bougatfa University Hospital (Bizerte), La Rabta University Hospital (Tunis) and Mohamed Tahar Maamouri University Hospital (Nabeul). Plasma samples were collected from each patient after obtaining written informed consent.

### NS5A amplification

Total RNA was extracted from 140 μl of plasma using the QIAamp viral RNA method (Qiagen, Courtaboeuf, France) according to the manufacturer’s recommendations. After reverse transcription (RT) using the SuperScript® III One-Step RT-PCR system with Platinum® Taq High Fidelity DNA Polymerase (Invitrogen, Cergy-Pontoise, France), a nested-polymerase chain reaction (nested-PCR) was performed with AmpliTaq Gold DNA Polymerase (Applied Biosystem, Courtaboeuf, France).

HCV RNA was amplified as previously described using primers 1a-NS5A-F0 (5’-GACATCTGGGACTGGATATGYGA -3’; nt 6276–6298) and 1a-NS5A-R0 (5’- GTCCAGGWRTARGACATYGAGCA-3’; nt 7599–7621) for subtype 1a and 1b-NS5A-F0 (5’- GAYGTTTGGGAYTGGATATGCAC-3’; nt 6276–6298) and 1b-NS5A-R0 (5’- GTCCAYGWRTARGACATYGAGCA -3’; nt 7599–7621) for subtype 1b. The PCR product obtained was 1345 bp in size [24]. Briefly, the PCR products in the first PCR round were obtained by using the following conditions: 30 min at 50 °C for the RT followed by denaturation at 94 °C for 10 min and then 40 cycles of amplification. Each cycle consisted of denaturation at 94 °C for 15 s, annealing at 60 °C for 30 s, and extension at 68 °C for 2 min. These steps were followed by an extension step at 68 °C for 10 min.

For the nested PCR, two overlapping fragments of DNA were amplified. For subtype 1a, the first fragment was amplified as previously described [24] using the primers 1a-NS5A-F1 (5’- GATATGYGAGGTGYTGAGCGA-3’; 6290–6310) and 1a-NS5A-SeqR (5’- AAGGAGTCCARRATCACCAC -3’; nt 7089–7108) and the second fragment was amplified using the primers 1a-NS5A-SeqF (5’- ARCTGTCYGCWCCATCTCTCAAGG-3’; nt 6955–6978) and 1a-NS5A-R0 the same primer used in the first PCR round.

For subtype 1b, the first fragment was amplified using the primers 1b-NS5A-F1 (5’-GATATGYACGGTGYTGAYTGA-3’; nt 6290–6310) and 1b-NS5A-SeqR (5’- AARGAGTCCARRATYACYAC -3’; nt 7089–7108) and the second fragment was amplified using the primers 1b-NS5A-SeqF (5’- ARCTGTCYGCWCCATCTCTCAAGG -3’; nt 6955–6978) and 1b-NS5A-R0 as previously described [24]. For both subtypes, the PCR products obtained were 818 bp and 666 bp in size for the first and second amplified fragments, respectively.

Two microliters from the first PCR reaction were used in the nested PCR. The amplification conditions were the same for both subtypes: initiation of denaturation at 95 °C for 10 min followed by 40 cycles consisting of denaturation at 95 °C for 30 s, primer annealing at 56 °C for 30 s, and primer extension at 72 °C for 2 min, followed by a final extension of 10 min at 72 °C. The PCR amplification products were visualized on 1.5 % agarose gel stained with ethidium bromide under UV transilluminator after electrophoresis.

### NS5A direct sequencing and sequence analysis

The nested PCR products were purified using the Microspin™ S-400 HR Columns, GE Healthcare, Buckinghamshire, UK) according to the manufacturer’s instructions. DNA sequencing was performed using the Big Dye® Terminator v 3.1 Cycle Sequencing Kit with a DXL 3500 DX sequencer (Appied Biosystem, Courtaboeuf, France). The same primers used in the two semi-nested PCR for the amplification of the NS5A region for both subtypes 1a and 1b were used for sequencing in this region.

Nucleotide sequences were aligned with reference sequences of different subtypes with SeqScape v 2.7 (Applied Biosystem, Courtaboeuf, France) and analyzed for the presence of previously identified mutations conferring resistance to NS5A inhibitors. GeneBank accession numbers for NS5A reference sequences are AF009606 for subtype 1a and AY045702 for genotype 1b. The threshold of nucleotide mixture detection during sequencing of samples is estimated to be around 20 %.

### Mutations conferring resistance to HCV NS5A inhibitors

NS5A sequences were screened for amino acid substitutions conferring resistance to NS5A inhibitors. For genotype 1b, sequences were screened for variants L31F/M/V, P32L and Y93C/H/N, which act as primary resistance mutations and L23F, R30Q and P58S, which act as secondary resistance substitutions, thus enhancing the resistance of primary mutations but not conferring resistance by themselves. For subtype 1a, sequences were screened for substitutions M28T/V, Q30H/E/R/K, L31M/V, P32L and Y93C/H/N, which act as primary resistance mutations [19, 24, 25, 27].

## Abbreviations

HCV: Hepatitis C Virus
NS: Non-Structural
DAAs: Direct-Acting Antivirals
PEG-IFNα: Pegylated alpha Interferon
RBV: Ribavirin
SVR: Sustained Virologic Response
DCV: Daclatasvir
LPV: Ledipasvir
OBV: Ombitasvir
SOF: Sofosbuvir
HBV: Hepatitis B Virus
HIV: Human immunodeficiency virus
RNA: Ribonucleic Acid
RT: Reverse Transcription
PCR: Polymerase Chain Reaction
NT: Nucleotide
DNA: Deoxyribonucleic Acid
